# Cerebellar Oscillatory Patterns in Essential Tremor: Modulatory Effects of VIM-DBS

**DOI:** 10.1007/s12311-025-01787-1

**Published:** 2025-02-01

**Authors:** Taylor J. Bosch, Christopher Groth, Arturo I. Espinoza, Vishal Bharmauria, Oliver Flouty, Arun Singh

**Affiliations:** 1 Department of Psychology, University of South Dakota, Vermillion, SD USA; 2 Center for Brain and Behavior Research, University of South Dakota, Vermillion, SD USA; 3 Department of Neurology, University of Iowa, Iowa City, IA USA; 4 Department of Neurosurgery and Brain Repair, University of South Florida, Tampa, FL USA; 5 Division of Basic Biomedical Sciences, Sanford School of Medicine, University of South Dakota, 414 E. Clark St. Vermillion, Vermillion, SD 57069 USA; 6 Department of Neuroscience, Sanford School of Medicine, University of South Dakota, Sioux Falls, SD USA

**Keywords:** Ventral intermediate nucleus, Deep brain stimulation, Cerebellar, Oscillations, Essential tremor

## Abstract

Essential tremor (ET) is a common movement disorder, and while ventral intermediate nucleus deep brain stimulation (VIM-DBS) is a well-established treatment, its precise mechanisms or modulatory effects, particularly in relation to cerebellar oscillations, remain unclear. In this study, we hypothesized that VIM-DBS would modulate cerebellar oscillatory activity across both resting and motor task conditions, reflecting its impact on cerebello-thalamic pathways. Ten patients diagnosed with ET participated in this study. We examined the effects of VIM-DBS on mid-cerebellar oscillations during resting-state and lower-limb pedaling motor tasks. Frequency analysis was conducted on the resting-state signal and time-frequency analysis was performed on motor task-related signals. We explored the modulatory effects of VIM-DBS on oscillatory activity across delta, theta, alpha, beta, and gamma frequency bands. We found that ON VIM-DBS increased mid-cerebellar relative theta power during resting-state conditions, with no significant changes in other frequency bands. During a pedaling motor task, VIM-DBS led to significant reductions in theta, alpha, and gamma power, highlighting the frequency-specific effects of stimulation. VIM-DBS also increased peak acceleration of leg movements during the pedaling task. Furthermore, VIM-DBS selectively increased mid-frontal relative theta and beta power as well as mid-occipital relative theta power during resting condition, suggesting localized mid-cerebellar modulation. Moreover, similarity analyses between mid-cerebellar and nearby mid-occipital signals revealed differences in coherence, phase coherence, and cross-spectrum phase coherence. Overall, these results support the role of VIM-DBS in modulating mid-cerebellar oscillations in ET and provide new insights into the neural mechanisms underlying DBS efficacy.

**Supplementary Information:**

The online version contains supplementary material available at 10.1007/s12311-025-01787-1.

## Introduction

Essential tremor (ET) is one of the most common neurological diseases and is characterized by involuntary, rhythmic tremors that primarily affect the hands, head, and voice during voluntary movements or when maintaining a posture [1]. Although ET is one of the most frequent movement disorders, the exact pathophysiological mechanisms are not fully clear. Evidence in the literature has implicated the cerebellar-thalamo-cortical network as a very crucial pathway in the generation and propagation of these tremors [2, 3]. More specifically, it is believed that abnormal oscillations within this network can cause motor symptoms in ET [3–6]. The cerebellum plays a fundamental role in coordinating voluntary motor activity, maintaining balance, and ensuring smooth and accurate movements. It achieves these functions through complex interactions with the thalamus and cerebral cortex. The cerebellar-thalamo-cortical pathways are essential for integrating sensory and motor information and for fine-tuning motor output [7]. Disruptions or alterations in the oscillatory activity within these pathways can lead to motor dysfunctions, as observed in ET. Interestingly, our previous studies have observed abnormal oscillatory activities in the cerebellar region during resting, cognitive, and motor task conditions in people with Parkinson’s disease (PD), particularly in the theta (4–7 Hz) and beta (13–30 Hz) frequency ranges, suggesting that these abnormal oscillations contribute to motor and non-motor dysfunctions in PD [8–10]. Therefore, studying cerebellar oscillations can offer a promising direction for understanding the pathophysiology of ET.

Deep brain stimulation (DBS) has emerged as an effective treatment for different movement disorders, including ET. DBS of the ventral intermediate nucleus (VIM) of the thalamus is particularly effective in alleviating tremor symptoms in ET patients [11–13]. However, the precise mechanisms by which VIM-DBS exerts its therapeutic effects remain uncertain. It has been suggested that VIM-DBS modulates abnormal activity within the cerebellar-thalamocortical pathway, thereby restoring more normal motor control [14–16].

Neuroimaging studies in ET have provided significant insights into the effects of VIM-DBS on cerebellar structure and functional connectivity [17]. Structural MRI studies have shown that ET patients often exhibit cerebellar atrophy and other morphological changes, suggesting a primary role for the cerebellum in the pathophysiology of ET [18]. Functional MRI (fMRI) and positron emission tomography (PET) studies have further elucidated the impact of VIM-DBS on cerebellar activity and connectivity [19, 20]. These studies demonstrate that VIM-DBS can modulate cerebellar activity, resulting in changes in functional connectivity within the cerebellar-thalamo-cortical network. Additionally, VIM-DBS has been shown to influence cerebellar blood flow and metabolism, as evidenced by PET studies, suggesting a direct impact on cerebellar function [21]. These neuroimaging findings collectively support the concept that VIM-DBS not only alleviates tremor symptoms but also induces significant neurophysiological changes in cerebellar structure and functional connectivity, thereby offering a mechanistic understanding of its therapeutic effects in ET. Furthermore, investigating how VIM-DBS affects cerebellar oscillations can provide critical insights into its mode of action and the underlying neurophysiological changes in ET.

In this study, we explored the cerebellar oscillations in ET patients by recording cerebellar electroencephalography (EEG) in both OFF- and ON VIM-DBS conditions. We assessed these oscillations during resting-state and lower-limb movement tasks. The resting-state condition was included to establish a baseline measure of cerebellar oscillatory activity without task-related influences. Resting-state oscillations can reveal the intrinsic properties of the cerebellar network and its baseline functional state. The lower-limb movement task was designed to directly engage the motor aspects of the cerebellar-thalamo-cortical network. This task allowed us to examine how VIM-DBS modulates cerebellar oscillations during active motor execution. The pedaling task was specifically chosen to minimize tremor-related confounds in motor kinematics. Unlike tasks involving upper-limb or tremor-affected movements, pedaling provides a consistent and tremor-independent movement framework, ensuring that observed differences are more likely attributable to the effects of DBS rather than variations in tremor severity. Understanding the effects of VIM-DBS on oscillatory activity during motor tasks can provide insights into the mechanisms by which DBS improves motor symptoms in ET [11, 13, 17, 22].

We hypothesized that VIM-DBS-induced changes in cerebellar oscillatory activity would be observed across both resting and motor task conditions. These modulations may further influence thalamo-cerebellar pathways, contributing to the therapeutic impact of VIM-DBS in improving motor function in ET patients. Given the paucity of prior research on VIM-DBS mechanisms in ET, this study adopts an exploratory approach to comprehensively analyze oscillatory activity across frequency bands during both tasks. These neuromodulatory changes may provide valuable insights into the role of cerebellar oscillations in the pathophysiology of ET, as well as the therapeutic mechanisms underlying VIM-DBS. In this study, we aim at advancing understanding of the pathophysiology of ET, particularly from the perspective of how DBS of the VIM interacts with cerebellar oscillations and improving development of targeted interventions for this debilitating condition.

## Materials and Methods

We recruited 10 ET subjects with bilateral VIM-DBS electrodes for this study. All participants provided their written informed consent, and procedures were approved by the University of Iowa Institutional Review Board in accordance with the declaration of Helsinki. Participants received VIM-DBS at either a therapeutic high frequency (130–190 Hz, ON VIM-DBS) or 0 Hz (OFF-VIM-DBS). The order of stimulation was counterbalanced, with a 20-minute washout period between types to mitigate transient sensory effects. This waiting period ensured optimal ON VIM-DBS response by allowing any lingering effects from previous stimulation to dissipate. All other stimulation parameters, including electrode contacts, amplitude, and pulse width, remained consistent based on subjects’ individualized DBS settings. All patients were on their prescribed medication for ET during the testing day to maintain consistency with their clinical treatment regimen. All clinical details can be seen in Table 1.

**Table 1:** Clinical characteristics

Subject	Gender	Age	Disease Duration	Medications	Left VIM Settings	Right VIM Settings
1	Female	69	51	Propranolol 80 mg QID	2+/1–3.0 V, 60µs, 130 Hz	11+ /10-, 2.5 V, 60µs, 130 Hz
2	Female	71	16	Clonazepam 0.25 mg QD	3+/1-, 3.3 V, 60µs. 160 Hz	11+ /9-, 2.8 V, 60µs, 160 Hz
3	Female	78	19	Propranolol LA 160 mg QD and Primidone 200 mg QHS	3+/2 and 1-, 3.65 V, 60µs, 140 Hz	11+ /10- and 9-, 2.8 V, 60µs, 140 Hz
4	Female	75	16	none	Case +/ 1 -, 2.8 V, 60µs, 170 Hz	Case +/ 8 and 9-, 4.05 V, 60µs, 170 Hz
5	Male	62	6	none	Case +/ 1-, 1.55 V, 60µs, 160 Hz	8+ /9-, 1.0 V, 60µs, 150 Hz
6	Female	70	8	none	1+/ 2-, 2.8 V, 60µs, 220 Hz	9+ /10-, 2.2 V, 60µs, 220 Hz
7	Female	76	30	Gabapentin 100 mg TID, Topiramate 75 mg BID, Topiramate 50 mg QHS	3+/ 2-, 4.1 V, 60µs, 170 Hz	Case +/ 2-, 3.85 V, 60µs, 145hz
8	Male	59	24	none	Case +/ 2 and 1-, 3.5 V, 90µs, 160 Hz	2+ /1-, 0.9 V, 60µs, 130 Hz
9	Male	76	12	Clonazepam 0.5 mg PRN, Propranolol LA 120 mg BID	Case +/ 1-, 2.35 V, 60µs, 130 Hz	Case + /10-, 1.95 V, 60µs, 130 Hz
10	Female	46	21	Propranolol 20 mg QD and Gabapentin 300 mg QD	Case +/ 2-, 1.6 V, 60µs, 190 Hz	Case + /10-, 1.6 V, 60µs, 190 Hz

During the resting-state task, participants sat comfortably in a quiet, climate controlled, room with their eyes open for 180 s while EEG signals were collected. For the lower-limb pedaling task, participants were seated and instructed to complete one full rotation of a pedaling device after a visual ‘Go-Cue’ (green circle) appeared 1000–2000 ms after a ‘Warning-Cue’ (black circle, 500 ms). Subject stopped the pedaling in the starting position and waited 3 s (inter-trial interval) for the next ‘Go-Cue’. This task was completed in two blocks of either 30 or 50 trials. A three-axis accelerometer was attached to the left leg since the task was initiated from the left leg. Accelerometer signals were synchronized with EEG data. For the pedaling trials, peak acceleration from the accelerometer signal was extracted to quantify the kinematics of each trial, and the average peak acceleration across trials was used for analysis.

EEG signals during both tasks were recorded using a 64-channel cap at a 500 Hz sampling rate with a 0.1 Hz high-pass filter, using Pz as a reference. The cap included left (Cb1), right (Cb2), and mid-cerebellar (Cbz) electrodes positioned over the posterior fossa corresponding to cerebellar lobules VII, VIII, and IX. EEG signals were processed using EEGLAB toolbox [23]. Electrodes Fp1, Fp2, FT9, FT10, TP9, and TP10 were removed due to susceptibility to artifacts. Initially, DBS-induced artifacts were removed using the DBSFILT function [24], which includes temporal filtering, spike/artifact detection, and spike removal through interpolation. Temporal filtering was applied by combining a low-pass filter (94.5 Hz) and a high-pass filter (0.75 Hz) to restrict EEG activity to frequencies below the DBS stimulation frequency, effectively filtering out much of the DBS-induced activity. For spike detection, Hampel filtering was applied in the frequency domain [25], after which detected spikes were removed. This process significantly attenuated DBS-induced artifacts in the EEG signals.

Next, the resting data was segmented into 3-second epochs, while the pedaling data was divided into epochs spanning 1 s before and 3 s after the ‘Go-Cue’. To further clean the data, bad epochs and artifacts were removed using the FASTER algorithm [26] and the pop_rejchan function, and eye-blink artifacts were addressed through ICA. The EEG signals were then band-pass filtered (1–50 Hz) and re-referenced to the average.

In post-processing of the resting-state data, spectral analysis was performed on the epoched data using the Welch method with a 1-second window and 50% overlap. Relative power was calculated for each frequency band (frequency-specific power divided by total spectral power from 1 to 50 Hz) to account for inter-subject variability.

For the pedaling tasks, time-frequency analysis using Morlet wavelet transform was conducted on each trial, with each pedaling trial trimmed to a duration of -0.5 to + 2 seconds. Logarithmically spaced frequency bands between 1 and 50 Hz were selected, and power was normalized by converting it to a decibel (dB) scale. Baseline power for each frequency was calculated as the average power from − 300 to -200 ms before the onset of the ‘Go-Cue’ stimulus. This brief baseline period is standard in the field, as a short time sample captures the wavelet-weighted impact of extended time and frequency ranges. Analysis focused on the 0–2 s window for all frequency bands since it took almost 2 s to complete the one rotation of the pedaling task. Moreover, to quantify the time-frequency analyses, we established a size threshold for statistical cluster significance based on 1000 permutations.

To examine phase-specific effects during the pedaling task, we conducted an inter-trial phase coherence (ITPC) analysis to evaluate the consistency of oscillatory phase alignment across trials for ON and OFF DBS conditions. Time-frequency decomposition was applied to the epoched signals as previously described, and the phase angle for each frequency and time point was extracted. ITPC was then computed by calculating the magnitude of the average phase vector across trials, yielding values between 0 (indicating no phase consistency) and 1 (indicating perfect phase alignment).

To determine the effects of VIM-DBS on cerebellar oscillations during both resting-state and pedaling tasks, analyses were focused on the mid-cerebellar (Cbz) electrode. Comparisons were also made with the mid-frontal cortical (FCz) and nearby mid-occipital (Oz) electrodes. We extracted mean power values from the delta (1–4 Hz), theta (4–7 Hz), alpha (7–13 Hz), beta (13–30 Hz), and gamma (30–50 Hz) frequency bands for statistical comparisons in both resting and pedaling-task conditions. Pedaling task-related ITPC values were averaged within the 0–500 ms window after the Go cue, and statistical comparisons were made between OFF and ON DBS conditions for all five frequency bands.

We performed paired samples t-tests to compare mid-cerebellar oscillations at each frequency band between the OFF and ON states of VIM-DBS during both the resting-state and the lower-limb pedaling task. Additionally, we assessed the pedaling kinematics outcomes under these conditions. To rule out global effects of stimulation, we conducted repeated-measures analysis of variance (rmANOVA) on mid-frontal ‘FCz’ oscillations during resting-state. The rmANOVA included within-subjects factors for channel (Cbz, FCz) and stimulation state (OFF, ON). We examined mid-occipital ‘Oz’ signals in a similar manner for the resting-state data and then performed various similarity analyses: coherence estimates, phase coherence, and cross-spectrum phase analysis. These methods are crucial for signal similarity analyses as they provide insights into the relationships between signals over time. Coherence estimates measure the degree of synchronization between signals across specific frequency bands, while phase coherence assesses the consistency of phase relationships. Cross-spectrum phase analysis further elucidates the timing and phase relationships between signals, enabling a deeper understanding of neural connectivity and coordinated activity.

## Results

**Fig. 1:**
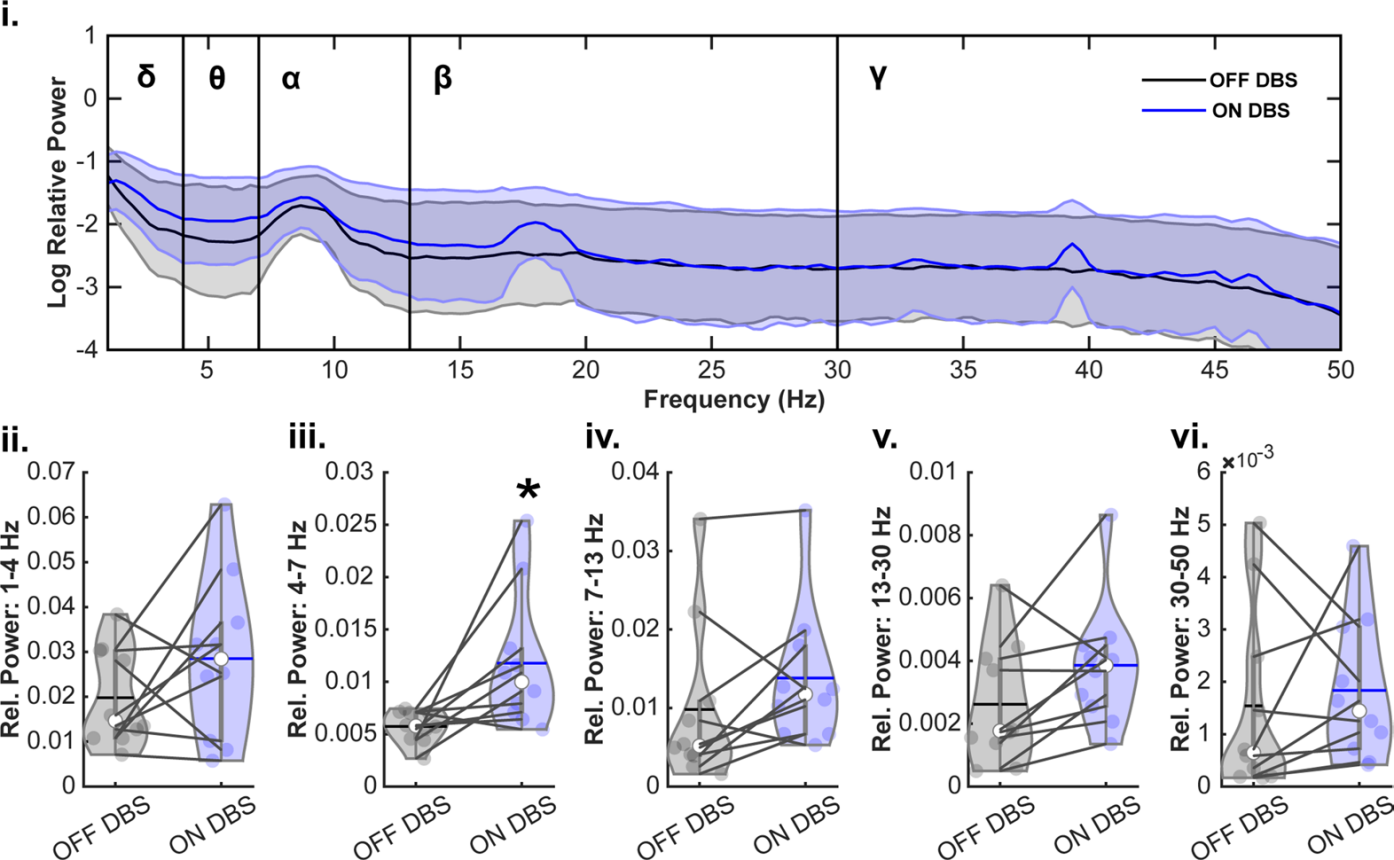
VIM-DBS modulates mid-cerebellar oscillations during resting-state. VIM-DBS resulted in a significant increase in theta band relative power (iii, 4–7 Hz), but differences were not seen in delta (ii, 1–4 Hz), alpha (iv, 7–13 Hz), beta (v, 13–30 Hz), or gamma (vi, 30–50 Hz) frequency bands. *p < 0.05 vs. OFF DBS. The horizontal lines and white circles in the violin plots represent the mean and median values, respectively

Initially, we examined whether ON VIM-DBS modulate mid-cerebellar ‘Cbz’ oscillations during the resting-state task across all frequency bands. When comparing OFF VIM-DBS versus ON VIM-DBS, we used paired samples t-tests and found that ON VIM-DBS resulted in significantly increased relative power in the theta frequency band (t_(9)_ = 2.81, *p* = 0.02). But we did not find significant differences between OFF VIM-DBS and ON VIM-DBS in delta (t_(9)_ = 1.43, *p* = 0.19), alpha (t_(9)_ = 0.098, *p* = 0.10), beta (t_(9)_ = 2.16, *p* = 0.06), or gamma (t_(9)_ = 0.55, *p* = 0.60) frequency bands (Fig. 1).

**Fig. 2:**
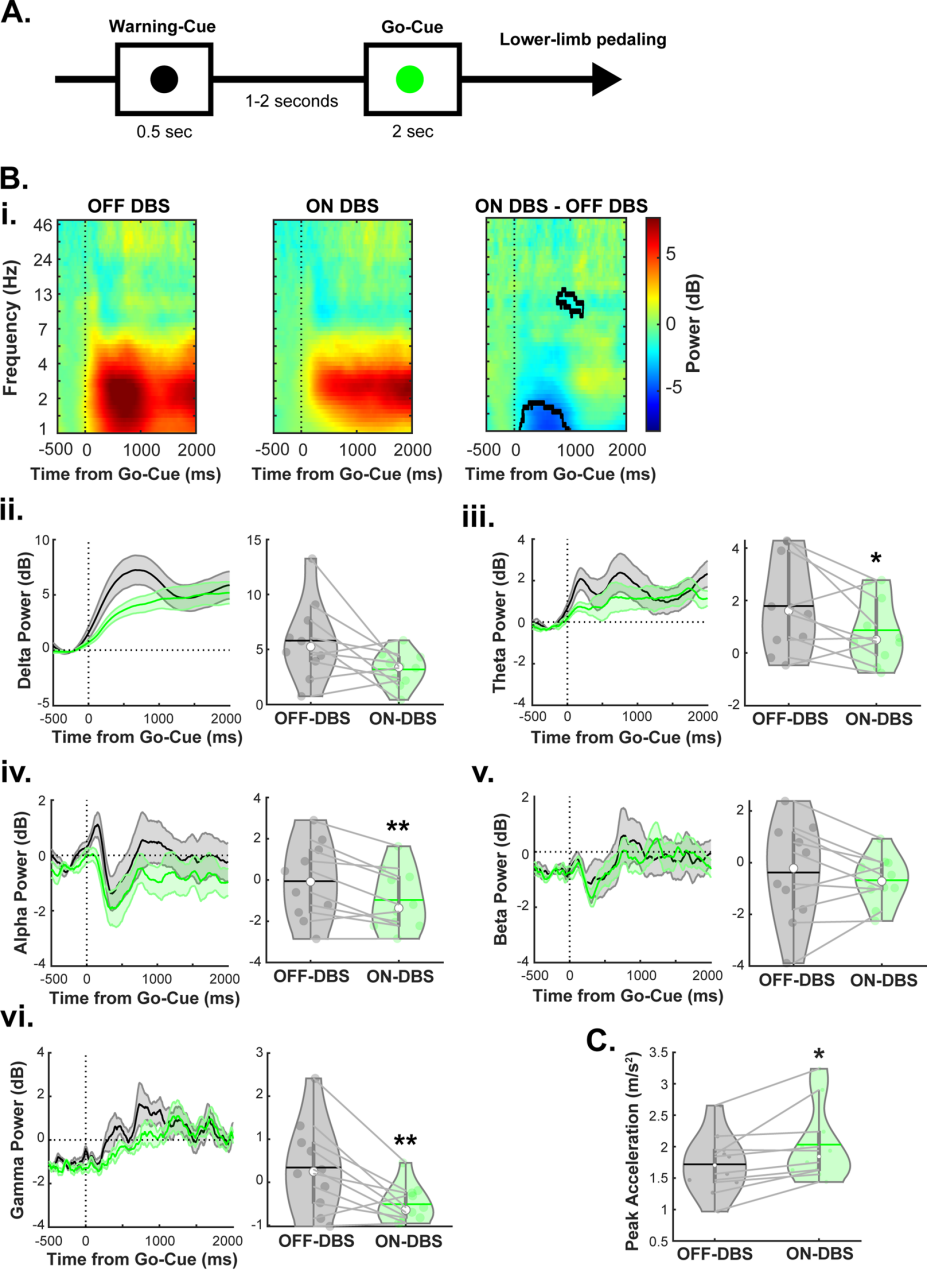
VIM-DBS modulates mid-cerebellar oscillations during a lower-limb pedaling motor task. **A**) Task protocol: participants were shown a ‘Go-Cue’ and completed one full rotation of the pedal 1–2 s after a ‘Warning-Cue’. **B**) Time-frequency plots (i), VIM-DBS resulted in decreased power in the theta (iii, 4–7 Hz), alpha (iv, 7–13 Hz), and gamma (vi, 30–50 Hz) frequency bands. No changes were observed in delta (ii, 1–4 Hz) or beta (v, 13–30 Hz) frequency bands. **C**) VIM-DBS also resulted in increases in peak acceleration during the pedaling task. *p < 0.05, **p < 0.01 vs. OFF-DBS. The horizontal lines and white circles in the violin plots represent the mean and median values, respectively

For the pedaling task (Fig. 2A), we examined whether ON VIM-DBS modulates mid-cerebellar EEG oscillations during the motor task. Compared to OFF VIM-DBS condition, we observed significantly decreased power in the theta (t_(9)_ = -2.23, *p* = 0.05), alpha (t_(9)_ = -3.74, *p* = 0.005), and gamma (t_(9)_ = -3.32, *p* = 0.01) frequency bands during the pedaling task in the ON VIM-DBS condition. No differences were observed in delta (t_(9)_ = -2.2, *p* = 0.06) or beta frequency (t_(9)_ = 0.77, *p* = 0.46) bands (Fig. 2B). Furthermore, when examining pedaling kinematics and effects of ON VIM-DBS, it was shown that ON VIM-DBS resulted in significantly increased peak acceleration (t_(9)_ = 3.18, *p* = 0.01) of leg movements (Fig. 2C). These results demonstrate that ON VIM-DBS can modulate mid-cerebellar oscillations during both resting as well as motor task conditions in patients with ET.

The ITPC analysis revealed significant differences between ON and OFF DBS conditions in specific frequency bands during the onset of the pedaling task (0–500 ms after the Go cue). In the delta band, ITPC was significantly reduced in the ON DBS condition compared to the OFF DBS condition (t_(9)_ = 2.37, *p* = 0.042, Fig. S1), suggesting decreased phase alignment of low-frequency oscillations during task initiation under DBS in ET patients. Similarly, in the alpha band, ITPC was also reduced in the ON DBS condition (t_(9)_ = 2.36, *p* = 0.042, Fig. S1), indicating that DBS influences neural phase dynamics related to motor preparatory or attentional processes. These findings suggest that DBS modulates phase coherence in the delta and alpha bands during pedaling motor task initiation, highlighting its impact on the temporal coordination of oscillatory activity in ET.

To ensure that there were no global effects of stimulation, we also examined the resting-state-related mid-frontal ‘FCz’ oscillations at each frequency band and compared with mid-cerebellar ‘Cbz’ oscillations (Fig. S2). The rmANOVA showed a main effect of channel location (Cbz and FCz: f_(1,9) =_ 6.8, *p* = 0.028), but no effect of stimulation (OFF and ON: f_(1,9) =_ 3.1, *p* = 0.11), or interaction (f_(1,9) =_ 0.21, *p* = 0.66) in the delta frequency band. In the theta frequency band, there was a main effect of channel location (f_(1,9)_ = 6.16, *p* = 0.035) and a main effect of stimulation (f_(1,9)_ = 6.1, *p* = 0.036), but there was no interaction (f_(1,9)_ = 1.64, *p* = 0.232). In the alpha frequency band, there was no main effect of channel (f_(1,9)_ = 3.48, *p* = 0.09), stimulation (f_(1,9)_ = 2.25, *p* = 0.17), or interaction (f_(1,9)_ = 1.94, *p* = 0.2). In the beta frequency band, there was a main effect of channel (f_(1,9)_ = 45.8, *p* < 0.001) and a main effect of stimulation (f_(1,9)_ = 9.05, *p* = 0.015), but no interaction (f_(1,9)_ = 0.43, *p* = 0.53). In the gamma frequency band, there was no main effect of channel (f_(1,9)_ = 1.71, *p* = 0.22, stimulation (f_(1,9)_ = 0.54, *p* = 0.48), or interaction (f_(1,9)_ = 0.06, *p* = 0.81). Given the results in the theta and beta frequency bands in the mid-frontal ‘FCz’ location, it is unlikely that results observed over mid-cerebellar ‘Cbz’ were the result of global stimulation.

Further, we examined nearby mid-occipital ‘Oz’ signals to determine the specificity of stimulation-induced resting-state oscillatory differences (Fig. S3). The rmANOVA test showed no main effect of channel (f_(1,9)_ = 1.48, *p* = 0.26), stimulation (f_(1,9)_ = 1.93, *p* = 0.2), or interaction (f_(1,9)_ = 0.84, *p* = 0.38) in the delta frequency band. In the theta frequency band, we observed no main effect of channel (f_(1,9)_ = 0.86, *p* = 0.38) but there was a main effect of stimulation (f_(1,9)_ = 11.12, *p* = 0.009). However, there was no interaction in theta band (f_(1,9)_ = 0.64, *p* = 0.44). There was no main effect of channel (f_(1,9)_ = 0.28, *p* = 0.61), stimulation (f_(1,9)_ = 3.45, *p* = 0.1), or interaction (f_(1,9)_ = 0.02, *p* = 0.89) in the alpha frequency band. We also observed no main effect of channel (f_(1,9)_ = 1.19, *p* = 0.3), stimulation (f_(1,9)_ = 4.27, *p* = 0.07), or interaction (f_(1,9)_ = 1.05, *p* = 0.33) in the beta frequency band, and no main effect of channel (f_(1,9)_ = 3.35, *p* = 0.1), stimulation (f_(1,9)_ = 0.09, *p* = 0.77), or interaction (f_(1,9)_ = 1.98, *p* = 0.19) in the gamma frequency band. Though these differences are similar, likely due to the close proximity of the electrodes, similarity analyses demonstrated that the signals exhibited differences in terms of coherence estimate, phase coherence, and cross-spectrum phase coherence (Fig. S4). Though the changes in spectral power were similar in the nearby mid-cerebellar and mid-occipital electrodes, there were notable differences in the level of synchronization between both signals (coherence estimate; Fig. S4A), the phase relationships between regions (phase coherence; Fig. S4B), and variations in coherence across frequencies (cross-spectrum phase; Fig. S4C).

## Discussion

In the current study, we investigated the modulatory effects of VIM-DBS on mid-cerebellar oscillations during a resting and lower-limb pedaling motor task conditions, with a focus on exploring changes across various frequency bands. Our first finding was that ON VIM-DBS significantly increased resting-state relative power in the theta frequency band compared to the OFF condition, suggesting that VIM-DBS can modulate mid-cerebellar oscillations specifically within the theta frequency range in the resting state. This increase in resting-state theta power indicates that VIM-DBS may influence functional connectivity between the thalamus and cerebellar regions potentially enhancing coordination and communication in circuits relevant for tremor suppression and motor control [22, 27]. However, we did not observe significant changes in other frequency bands, including delta, alpha, beta, or gamma, when comparing the effects of ON to the OFF VIM-DBS condition in the resting-state. These results indicate that the influence of VIM-DBS on cerebellar oscillatory activity may be selective to certain frequency bands, specifically the theta band, rather than inducing broad-spectrum changes across all oscillatory dynamics. The absence of significant modulation in the other frequency bands such as delta, alpha, and gamma frequency bands in the mid-cerebellar region could indicate that these frequencies may not be involved in the neural mechanisms targeted by VIM-DBS. While the beta band approached significance (*p* = 0.06), the results indicate only a trend toward increased power. This trend may reflect the role of beta oscillations in the cerebello-thalamocortical network, which has been implicated in motor control [28]. Beta oscillations are known to contribute to motor planning and execution, and modulation of mid-cerebellar beta activity by VIM-DBS could indicate an important functional role in the refinement of motor activity. However, further research with a larger sample size is needed to confirm this trend and better understand its implications.

The significant increase in resting-state theta power with ON VIM-DBS could imply a compensatory mechanism or direct modulation of cerebellar circuits. Theta oscillations are often associated with cognitive processes such as attention and memory in patients with movement disorders [29–31], suggesting that VIM-DBS might enhance these functions through its impact on cerebellar activity. Our recent study demonstrated modulation of cerebellar oscillations in the theta and beta frequency bands, which correlated with motor severity in PD patients following high-frequency subthalamic nucleus (STN) DBS, suggesting valuable insights into the complex interactions within the basal ganglia-thalamocortical circuits [32]. In addition, theta oscillations in the cortico-basal ganglia-cerebellar circuits are implicated in dystonia [33], which is often comorbid with ET. Although none of the patients in this study had comorbid dystonia, the role of these oscillations in dystonia-related motor dysfunction may provide valuable insights into the cerebellar theta power changes observed in ET. Future studies involving dystonia patients with comorbid tremor could further elucidate the shared mechanisms underlying these overlapping neural pathways. Overall, the selective increase in theta power indicates that VIM-DBS may exert frequency-specific effects on cerebellar activity during the resting condition, potentially aligning with its role in reducing tremors in ET patients via modulating cerebello-thalamic circuitry [34].

We further explored the effects of therapeutic high-frequency VIM-DBS on cerebellar EEG oscillations during a lower-limb pedaling motor task in ET patients. Our results indicate that ON VIM-DBS led to decreases in power across the theta, alpha, and gamma frequency bands during the pedaling task. These outcomes show that VIM-DBS not only modulates cerebellar activity during resting-state but also alters during active motor tasks. The significant reduction in theta and alpha power during the pedaling task with ON VIM-DBS reflects the role of the cerebellar region in sensorimotor integration and motor coordination [35, 36]. These outcomes suggest that therapies like lesions and high-frequency stimulation of the motor thalamus effectively reduce ET symptoms by modulating abnormal low-frequency synchronization in the cerebello-thalamocortical motor network, similar to the effects of DBS in other disorders [37, 38]. Decreased theta and alpha frequency band power during active movement in the mid-cerebellar area may suggest a reorganization of neural activity, promoting efficient motor execution in response to DBS. Previous studies have indicated that both theta and alpha oscillations are crucial for maintaining attention and coordination during motor tasks [29, 39], and the observed reductions in the mid-cerebellar oscillations may indicate enhanced motor performance through DBS-mediated modulation of these frequency bands in ET patients. Interestingly, the reduction in gamma power during the pedaling motor task aligns with previous findings, which demonstrate decreased gamma oscillations during DBS in motor control among patients with movement disorders [40]. Gamma activity has been associated with the fine-tuning of motor commands and movement precision. Its suppression during the pedaling task may reflect network-level adjustments facilitated by high-frequency VIM-DBS to optimize motor output in ET patients [41]. Beyond the effects on cerebellar oscillations, VIM-DBS also significantly increased peak acceleration during the pedaling task, demonstrating improved motor performance. This finding aligns with previous evidence suggesting that VIM-DBS enhances motor function in ET patients by modulating the cerebello-thalamo-cortical circuitry, which is critical for motor coordination and execution [22, 42]. To minimize tremor-related confounds in motor kinematics, this study utilized a lower-limb pedaling task to investigate DBS effects on cerebellar oscillatory activity. However, given that ET primarily affects the upper extremities, the lack of upper-limb motor task assessments, such as finger-tapping or tremor rating scales, represents a limitation of this work. Future studies incorporating these assessments will help better contextualize the functional relevance of VIM DBS in the clinical management of ET.

Taken together, these results provide strong evidence that VIM-DBS modulates cerebellar oscillations in a frequency-specific manner during both resting and active motor task conditions in ET patients. Future studies should further explore the relationship between changes in cerebellar oscillatory patterns and clinical improvements, such as motor performance and tremor severity, to better understand the therapeutic mechanisms underlying DBS. Additionally, examining the effects of VIM-DBS on cerebellar oscillations during other motor tasks or cognitive tasks may offer deeper insights into the broader functional impact of DBS in ET.

Furthermore, to confirm that the observed effects of VIM-DBS were localized to the mid-cerebellar region and not a result of global brain-wide changes, we analyzed oscillatory activity at the mid-frontal electrode across all frequency bands. The results indicated no significant global effects of stimulation, except for select findings in the theta and beta frequency bands. In the theta and beta frequency bands, we found an effect of stimulation, indicating that stimulation also influenced mid-frontal theta and beta oscillations. The lack of significant interactions or broad effects across other frequency bands in the mid-frontal region suggests that the modulation observed in the mid-cerebellar region was not driven by widespread cortical changes due to VIM-DBS. Notably, the isolated theta and beta frequency bands effects in the mid-frontal region are consistent with the selective involvement of theta and beta frequency bands in motor control, which may reflect indirect influences on motor networks [29, 43]. However, given the limited frontal involvement in delta, alpha, and gamma frequency bands, it is unlikely that the observed changes in the mid-cerebellar oscillations were the result of generalized stimulation effects across the cortical regions, similar to our previous studies [9, 32].

Moreover, in line with our earlier studies [8, 9, 32, 44], we analyzed mid-occipital ‘Oz’ signal to assess whether the modulatory effects of VIM-DBS on mid-cerebellar oscillations were specific or influenced by nearby mid-occipital region. Our results showed that VIM-DBS significantly increased resting-state relative power in the theta frequency band in the mid-occipital region, suggesting that VIM-DBS may influence oscillatory activity beyond the mid-cerebellar region. The selective increase in theta power in the mid-occipital region could reflect broader network effects of VIM-DBS, possibly through the modulation of cortico-cerebellar circuits that influence both motor and non-motor brain areas [45]. The absence of significant modulation in these other frequency bands (delta, alpha, beta, or gamma bands) suggests that the effects of VIM-DBS on the mid-occipital region are more limited compared to its impact on the cerebellum, further supporting the idea that DBS primarily targets cerebello-thalamic pathways. Moreover, rmANOVA results showed only the difference of stimulation in the theta band when both Oz and Cbz electrodes were compared. These findings suggest that while VIM-DBS exerts localized effects on mid-cerebellar oscillations, it may also produce selective changes in nearby regions, such as the occipital cortex, in the particular frequency band.

Although the mid-cerebellar and mid-occipital signals showed similar trends, likely attributable to the close proximity of the electrodes, further similarity analyses revealed important distinctions between the two regions. Specifically, differences emerged in measures of signal coherence, phase coherence, and cross-spectrum phase coherence [9, 32]. These findings suggest that, despite spatial proximity, mid-cerebellar and mid-occipital signals are functionally distinct and respond differently to VIM-DBS. The observed differences in coherence measures indicate that while both regions may experience some shared modulation, the nature of their oscillatory coupling and synchrony diverges under stimulation. Coherence estimates provide insight into the degree of linear synchronization between the two regions, and the variations in phase coherence and cross-spectrum phase coherence highlight differential timing and phase relationships between the mid-cerebellar ‘Cbz’ and mid-occipital ‘Oz’ signals. These distinctions suggest that VIM-DBS may influence cerebellar-thalamic and cortico-cerebellar networks differently, depending on the specific neural circuits involved in motor and non-motor functions.

Although all patients showed subjective improvement in tremor symptoms during ON DBS with therapeutic high-frequency parameters, we did not collect quantitative tremor control data to confirm a direct relationship between clinical improvement and cerebellar oscillatory changes. This represents a limitation of the current study. Future research involving larger cohorts and quantitative measures of clinical efficacy will be necessary to elucidate the link between mid-cerebellar oscillatory changes and tremor improvement. Overall, these findings support the conclusion that VIM-DBS has localized effects on mid-cerebellar oscillations, particularly in the specific frequency bands, as seen during both resting-state and lower-limb motor tasks, with minimal impact on global mid-frontal or mid-occipital oscillatory activity. This supports the specificity of VIM-DBS in modulating the cerebello-thalamic networks, thereby improving motor abnormalities in ET patients.

## Electronic Supplementary Material

Below is the link to the electronic supplementary material.

Supplementary Material 1

## Data Availability

All raw data and codes are available from the corresponding author upon reasonable request.

## References

[1] Benito-Leon J, Louis ED. Clinical update: diagnosis and treatment of essential tremor. Lancet. 2007;369:1152–4. 17416247 10.1016/S0140-6736(07)60544-3

[2] Troster AI, Woods SP, Fields JA, Lyons KE, Pahwa R, Higginson CI, Koller WC. Neuropsychological deficits in essential tremor: an expression of cerebello-thalamo-cortical pathophysiology? Eur J Neurol. 2002;9:143–51. 11882055 10.1046/j.1468-1331.2002.00341.x

[3] Woodward K, Apps R, Goodfellow M, Cerminara NL. Cerebello-Thalamo-Cortical Network Dynamics in the Harmaline Rodent Model of essential tremor. Front Syst Neurosci. 2022;16:899446. 35965995 10.3389/fnsys.2022.899446 PMC9365993

[4] Lee J, Kim J, Cortez J, Chang SY. Thalamo-cortical network is associated with harmaline-induced tremor in rodent model. Exp Neurol. 2022;358:114210. 36007599 10.1016/j.expneurol.2022.114210

[5] Pan MK, Kuo SH. Essential tremor: clinical perspectives and pathophysiology. J Neurol Sci. 2022;435:120198. 35299120 10.1016/j.jns.2022.120198 PMC10363990

[6] Wong SB, Wang YM, Lin CC, Geng SK, Vanegas-Arroyave N, Pullman SL, Kuo SH, Pan MK. Cerebellar oscillations in familial and sporadic essential tremor. Cerebellum. 2022;21:425–31. 34341893 10.1007/s12311-021-01309-9 PMC8970339

[7] Louis ED, Faust PL. Essential tremor pathology: neurodegeneration and reorganization of neuronal connections. Nat Rev Neurol. 2020;16:69–83. 31959938 10.1038/s41582-019-0302-1

[8] Bosch TJ, Espinoza AI, Singh A. Cerebellar oscillatory dysfunction during lower-limb movement in Parkinson’s disease with freezing of gait. Brain Res. 2023;1808:148334. 36931582 10.1016/j.brainres.2023.148334

[9] Bosch TJ, Groth C, Eldridge TA, Gnimpieba EZ, Baugh LA, Singh A. Altered cerebellar oscillations in Parkinson’s Disease patients during cognitive and motor tasks. Neuroscience. 2021;475:185–96. 34455014 10.1016/j.neuroscience.2021.08.021

[10] Bosch TJ, Groth C, Singh A. Resting-state low-frequency cerebellar oscillations can be abnormal in Parkinson’s Disease. Cerebellum. 2022;21:1139–43. 34755280 10.1007/s12311-021-01343-7

[11] Blomstedt P, Hariz GM, Hariz MI, Koskinen LO. Thalamic deep brain stimulation in the treatment of essential tremor: a long-term follow-up. Br J Neurosurg. 2007;21:504–9. 17922323 10.1080/02688690701552278

[12] Lozano AM. Vim thalamic stimulation for tremor. Arch Med Res. 2000;31:266–9. 11036177 10.1016/s0188-4409(00)00081-3

[13] Wang YM, Liu CW, Chen SY, Lu LY, Liu WC, Wang JH, Ni CL, Wong SB, Kumar A, Lee JC, Kuo SH, Wu SC, Pan MK. Neuronal population activity in the olivocerebellum encodes the frequency of essential tremor in mice and patients. Sci Transl Med. 2024;16:eadl1408. 38748772 10.1126/scitranslmed.adl1408

[14] Voegtle A, Terzic L, Farahat A, Hartong N, Galazky I, Hinrichs H, Nasuto SJ, de Oliveira Andrade A, Knight RT, Ivry RB, Voges J, Deliano M, Buentjen L, Sweeney-Reed CM. Ventrointermediate thalamic stimulation improves motor learning in humans. Commun Biol. 2024;7:798. 38956172 10.1038/s42003-024-06462-5 PMC11220095

[15] Gibson WS, Jo HJ, Testini P, Cho S, Felmlee JP, Welker KM, Klassen BT, Min HK, Lee KH. Functional correlates of the therapeutic and adverse effects evoked by thalamic stimulation for essential tremor. Brain. 2016;139:2198–210. 27329768 10.1093/brain/aww145 PMC4958905

[16] Tai CH, Tseng SH. Cerebellar deep brain stimulation for movement disorders. Neurobiol Dis. 2022;175:105899. 10.1016/j.nbd.2022.105899 36265768

[17] Madelein van der Stouwe AM, Nieuwhof F and Helmich RC. Tremor pathophysiology: lessons from neuroimaging. Curr Opin Neurol. 2020;33:474–81. 10.1097/WCO.0000000000000829 32657888

[18] Cerasa A, Quattrone A. Linking Essential Tremor to the Cerebellum-Neuroimaging Evidence. Cerebellum. 2016;15:263–75. 10.1007/s12311-015-0739-8 26626626

[19] Sharifi S, Nederveen AJ, Booij J, van Rootselaar AF. Neuroimaging essentials in essential tremor: a systematic review. Neuroimage Clin. 2014;5:217–31. 10.1016/j.nicl.2014.05.003 PMC4110352 25068111

[20] Wong JK, Hess CW, Almeida L, Middlebrooks EH, Christou EA, Patrick EE, Shukla AW, Foote KD, Okun MS. Deep brain stimulation in essential tremor: targets, technology, and a comprehensive review of clinical outcomes. Expert Rev Neurother. 2020;20:319–31. 10.1080/14737175.2020.1737017 PMC7174089 32116065

[21] Ceballos-Baumann AO, Boecker H, Fogel W, Alesch F, Bartenstein P, Conrad B, Diederich N, von Falkenhayn I,Moringlane JR, Schwaiger M, Tronnier VM. Thalamic stimulation for essential tremor activates motor and deactivates vestibular cortex. Neurology. 2001;56:1347–54. 10.1212/wnl.56.10.1347 11376186

[22] Neudorfer C, Kultas-Ilinsky K, Ilinsky I, Paschen S, Helmers AK, Cosgrove GR, Richardson RM, Horn A, Deuschl G. The role of the motor thalamus in deep brain stimulation for essential tremor. Neurotherapeutics. 2024;21:e00313. 10.1016/j.neurot.2023.e00313 PMC11103222 38195310

[23] Delorme A, Makeig S. EEGLAB: an open source toolbox for analysis of single-trial EEG dynamics including independent component analysis. J Neurosci Methods. 2004;134:9–21. 10.1016/j.jneumeth.2003.10.009 15102499

[24] Lio G, Thobois S, Ballanger B, Lau B, Boulinguez P. Removing deep brain stimulation artifacts from the electroencephalogram: Issues, recommendations and an open-source toolbox. Clin Neurophysiol. 2018;129:2170–85. 10.1016/j.clinph.2018.07.023 30144660

[25] Allen DP, Stegemoller EL, Zadikoff C, Rosenow JM, Mackinnon CD. Suppression of deep brain stimulation artifacts from the electroencephalogram by frequency-domain Hampel filtering. Clin Neurophysiol. 2010;121:1227–32. 10.1016/j.clinph.2010.02.156 PMC2924738 20362499

[26] Nolan H, Whelan R, Reilly RB. FASTER: Fully Automated Statistical Thresholding for EEG artifact Rejection. J Neurosci Methods. 2010;192:152–62. 10.1016/j.jneumeth.2010.07.015 20654646

[27] Fenoy AJ, Chu ZD, Ritter RJ, 3rd, Conner CR, Kralik SF. Evaluating functional connectivity differences between DBS ON/OFF states in essential tremor. Neurotherapeutics. 2024;21:e00375. 10.1016/j.neurot.2024.e00375 PMC11301224 38824101

[28] Basha D, Kalia SK, Hodaie M, Lopez Rios AL, Lozano AM, Hutchison WD. Beta band oscillations in the motor thalamus are modulated by visuomotor coordination in essential tremor patients. Front Hum Neurosci. 2023;17:1082196. 10.3389/fnhum.2023.1082196 PMC10169705 37180551

[29] Singh A, Cole RC, Espinoza AI, Brown D, Cavanagh JF, Narayanan NS. Frontal theta and beta oscillations during lower-limb movement in Parkinson’s disease. Clin Neurophysiol. 2020;131:694–702. 10.1016/j.clinph.2019.12.399 PMC8112379 31991312

[30] Singh A, Richardson SP, Narayanan N, Cavanagh JF. Mid-frontal theta activity is diminished during cognitive control in Parkinson’s disease. Neuropsychologia. 2018;117:113–22. 10.1016/j.neuropsychologia.2018.05.020 PMC6524769 29802866

[31] Singh A, Cole RC, Espinoza AI, Wessel JR, Cavanagh JF, Narayanan NS. Evoked mid-frontal activity predicts cognitive dysfunction in Parkinson’s disease. J Neurol Neurosurg Psychiatry. 2023;94:945–53. 10.1136/jnnp-2022-330154 PMC10592174 37263767

[32] Bosch TJ, Cole RC, Vuong SM, Flouty O, Singh A. Modulation of Cerebellar Oscillations with Subthalamic Stimulation in Patients with Parkinson’s Disease. J Parkinsons Dis. 2024;14:1417–26. 10.3233/JPD-240065 PMC11492035 39331106

[33] Neumann WJ, Jha A, Bock A, Huebl J, Horn A, Schneider GH, Sander TH, Litvak V, Kuhn AA. Cortico-pallidal oscillatory connectivity in patients with dystonia. Brain. 2015;138:1894–906. 10.1093/brain/awv109 25935723

[34] Milosevic L, Kalia SK, Hodaie M, Lozano AM, Popovic MR, Hutchison WD. Physiological mechanisms of thalamic ventral intermediate nucleus stimulation for tremor suppression. Brain. 2018;141:2142–55. 10.1093/brain/awy139 PMC6022553 29878147

[35] Caplan JB, Madsen JR, Schulze-Bonhage A, Aschenbrenner-Scheibe R, Newman EL, Kahana MJ. Human theta oscillations related to sensorimotor integration and spatial learning. J Neurosci. 2003;23:4726–36. 10.1523/JNEUROSCI.23-11-04726.2003 PMC6740775 12805312

[36] Babiloni C, Del Percio C, Arendt-Nielsen L, Soricelli A, Romani GL, Rossini PM, Capotosto P. Cortical EEG alpha rhythms reflect task-specific somatosensory and motor interactions in humans. Clin Neurophysiol. 2014;125:1936–45. 10.1016/j.clinph.2014.04.021 24929901

[37] Kane A, Hutchison WD, Hodaie M, Lozano AM, Dostrovsky JO. Enhanced synchronization of thalamic theta band local field potentials in patients with essential tremor. Exp Neurol. 2009;217:171–6. 10.1016/j.expneurol.2009.02.005 19233174

[38] Miocinovic S, Parent M, Butson CR, Hahn PJ, Russo GS, Vitek JL, McIntyre CC. Computational analysis of subthalamic nucleus and lenticular fasciculus activation during therapeutic deep brain stimulation. J Neurophysiol. 2006;96:1569–80. 10.1152/jn.00305.2006 16738214

[39] Tan E, Troller-Renfree SV, Morales S, Buzzell GA, McSweeney M, Antunez M, Fox NA. Theta activity and cognitive functioning: Integrating evidence from resting-state and task-related developmental electroencephalography (EEG) research. Dev Cogn Neurosci. 2024;67:101404. 10.1016/j.dcn.2024.101404 PMC11214181 38852382

[40] Wiest C, Tinkhauser G, Pogosyan A, He S, Baig F, Morgante F, Mostofi A, Pereira EA, Tan H, Brown P, Torrecillos F. Subthalamic deep brain stimulation induces finely-tuned gamma oscillations in the absence of levodopa. Neurobiol Dis. 2021;152:105287. 10.1016/j.nbd.2021.105287 PMC7116781 33549721

[41] Groppa S, Herzog J, Falk D, Riedel C, Deuschl G, Volkmann J. Physiological and anatomical decomposition of subthalamic neurostimulation effects in essential tremor. Brain. 2014;137:109–21. 10.1093/brain/awt304 24277721

[42] Bai Y, Yin Z, Diao Y, Hu T, Yang A, Meng F, Zhang J. Loss of long-term benefit from VIM-DBS in essential tremor: A secondary analysis of repeated easurements. CNS Neurosci Ther. 2022;28:279–88. 10.1111/cns.13770 PMC8739044 34866345

[43] Singh A. Oscillatory activity in the cortico-basal ganglia-thalamic neural circuits in Parkinson’s disease. Eur J Neurosci. 2018;48:2869–78. 10.1111/ejn.13853 29381817

[44] Bosch TJ, Kammermeier S, Groth C, Leedom M, Hanson EK, Berg-Poppe P and Singh A. Cortical and Cerebellar Oscillatory Responses to Postural Instability in Parkinson's Disease. Front Neurol. 2021;12:752271. 10.3389/fneur.2021.752271 PMC8599431 34803888

[45] Graff-Radford J, Foote KD, Mikos AE, Bowers D, Fernandez HH, Rosado CA, Rodriguez RL, Malaty IA, Haq IU, Jacobson CE, Okun MS. Mood and motor effects of thalamic deep brain stimulation surgery for essential tremor. Eur J Neurol. 2010;17:1040–6. 10.1111/j.1468-1331.2010.02958.x 20113336

