# Supplementary material for: Cerebellar Oscillatory Patterns in Essential Tremor: Modulatory Effects of VIM-DBS
Source: Cerebellum. 2025 Feb 1;24(2):40. doi: 10.1007/s12311-025-01787-1 (PMC11787153; doi:10.1007/s12311-025-01787-1)
Supplement: Supplementary file 1 — Supplementary Material 1 [file 12311_2025_1787_MOESM1_ESM.pdf]

Supplementary Materials

Cerebellar oscillatory patterns in essential tremor: modulatory effects of VIM-DBS

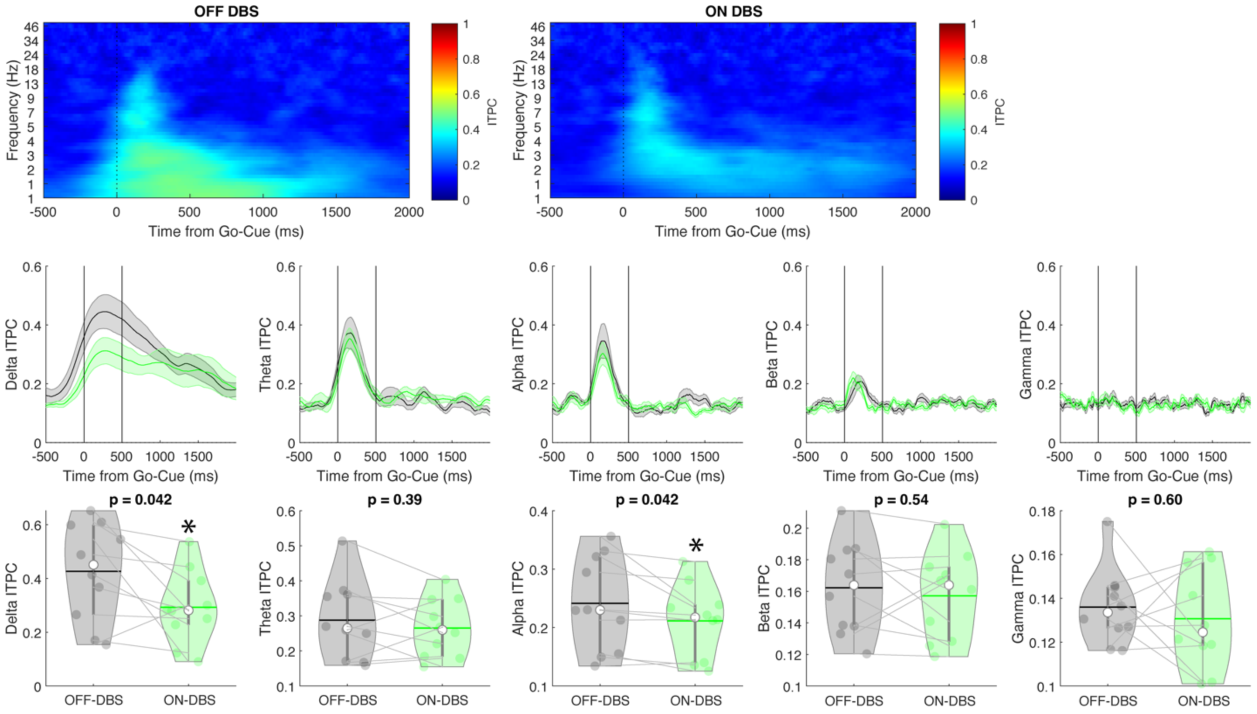

Fig. S1. Inter-trial phase coherence (ITPC) analysis. VIM-DBS modulates ITPC in the delta and alpha frequency bands within the mid-cerebellar region during a lower-limb pedaling motor task, highlighting the impact of DBS on cerebellar phase synchrony.

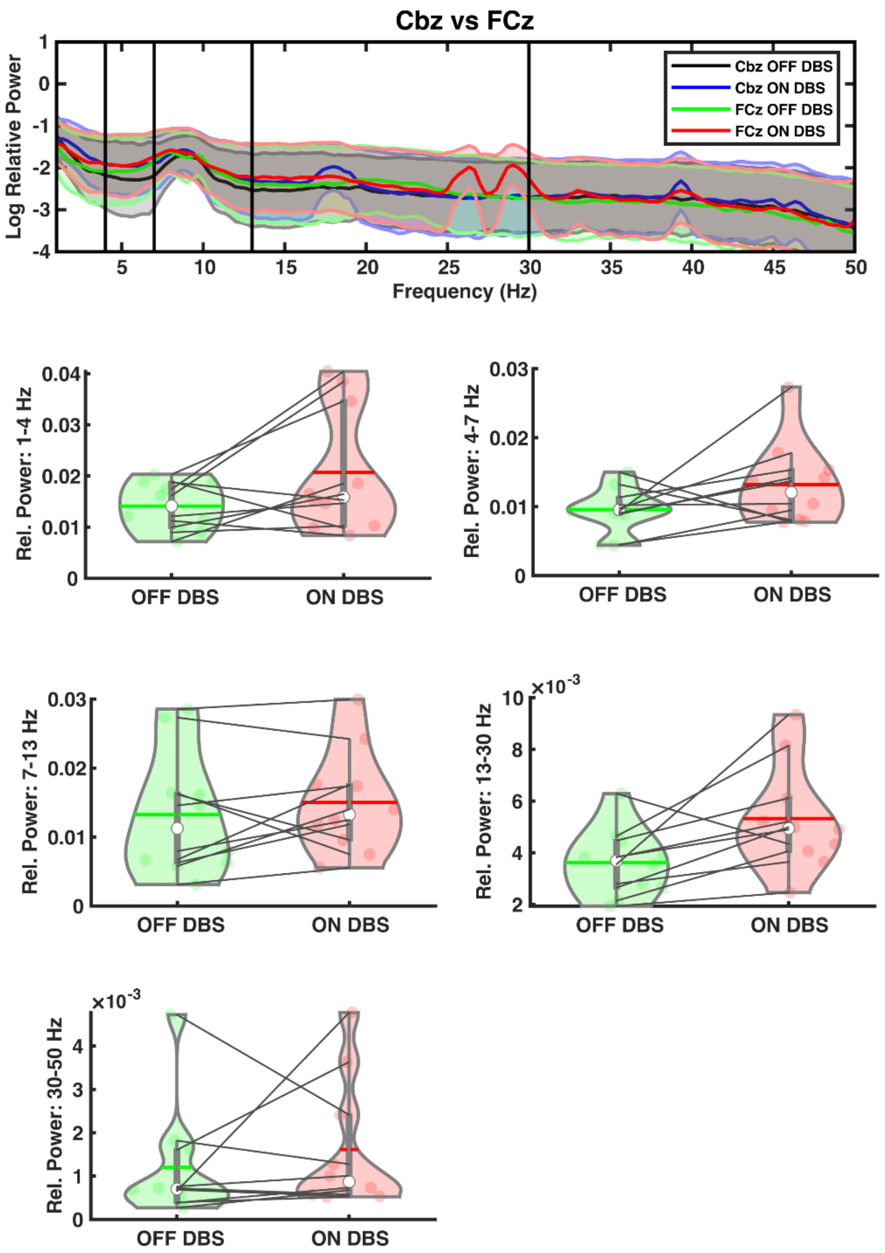

Fig. S2. Mid-frontal 'FCz' changes following VIM-DBS during the resting-state task. VIM-DBS resulted in increased theta (4-7 Hz) and beta (13-30 Hz) relative power values. No changes were seen across delta (1-4 Hz), alpha (7-13 Hz), or gamma (30-50 Hz) frequency bands.

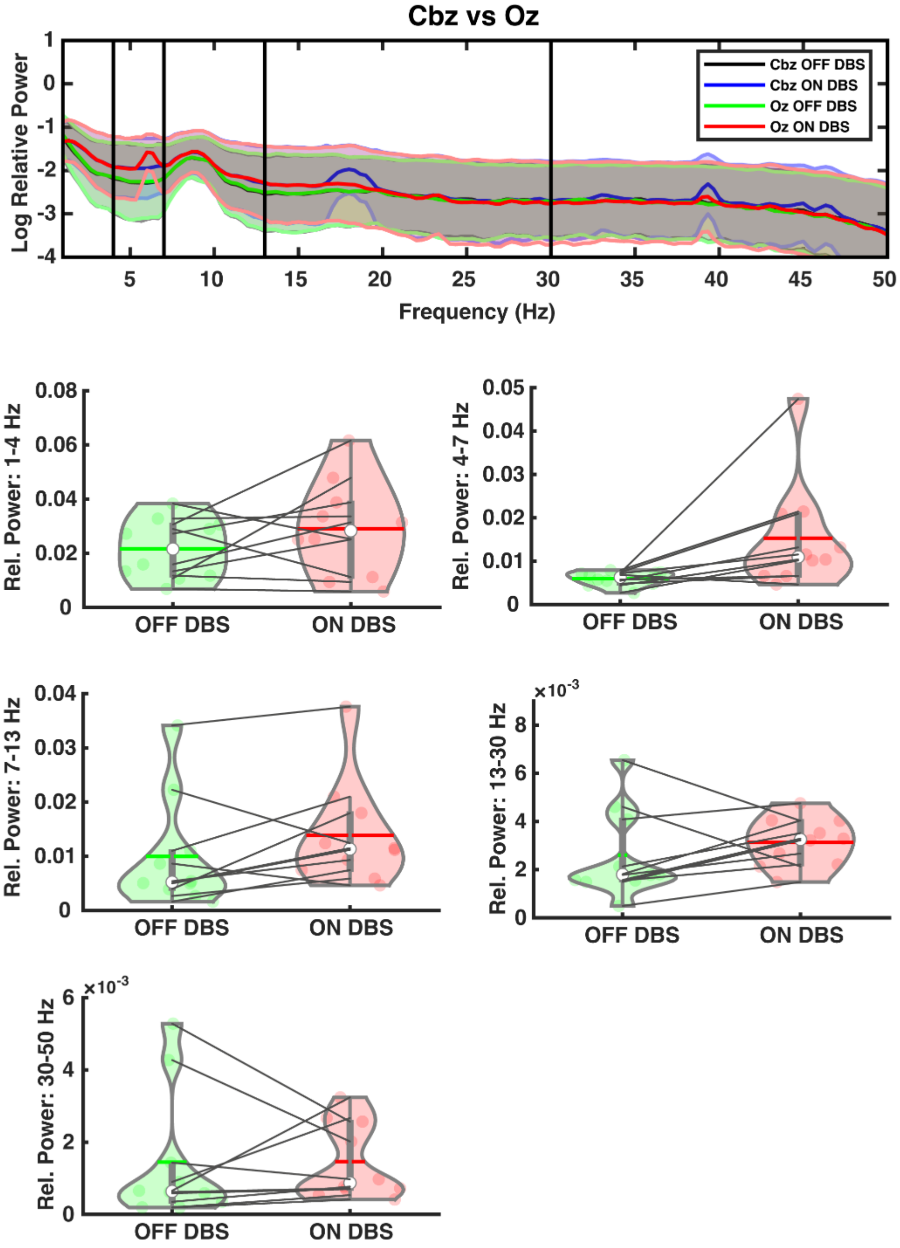

Fig. S3. Mid-occipital 'Oz' changes following VIM-DBS during the resting-state task. VIM-DBS resulted in increased theta (4-7 Hz) relative power value. No changes were seen across delta (1-4 Hz), alpha (7-13 Hz), beta (13-30 Hz), or gamma (30-50 Hz) frequency bands.

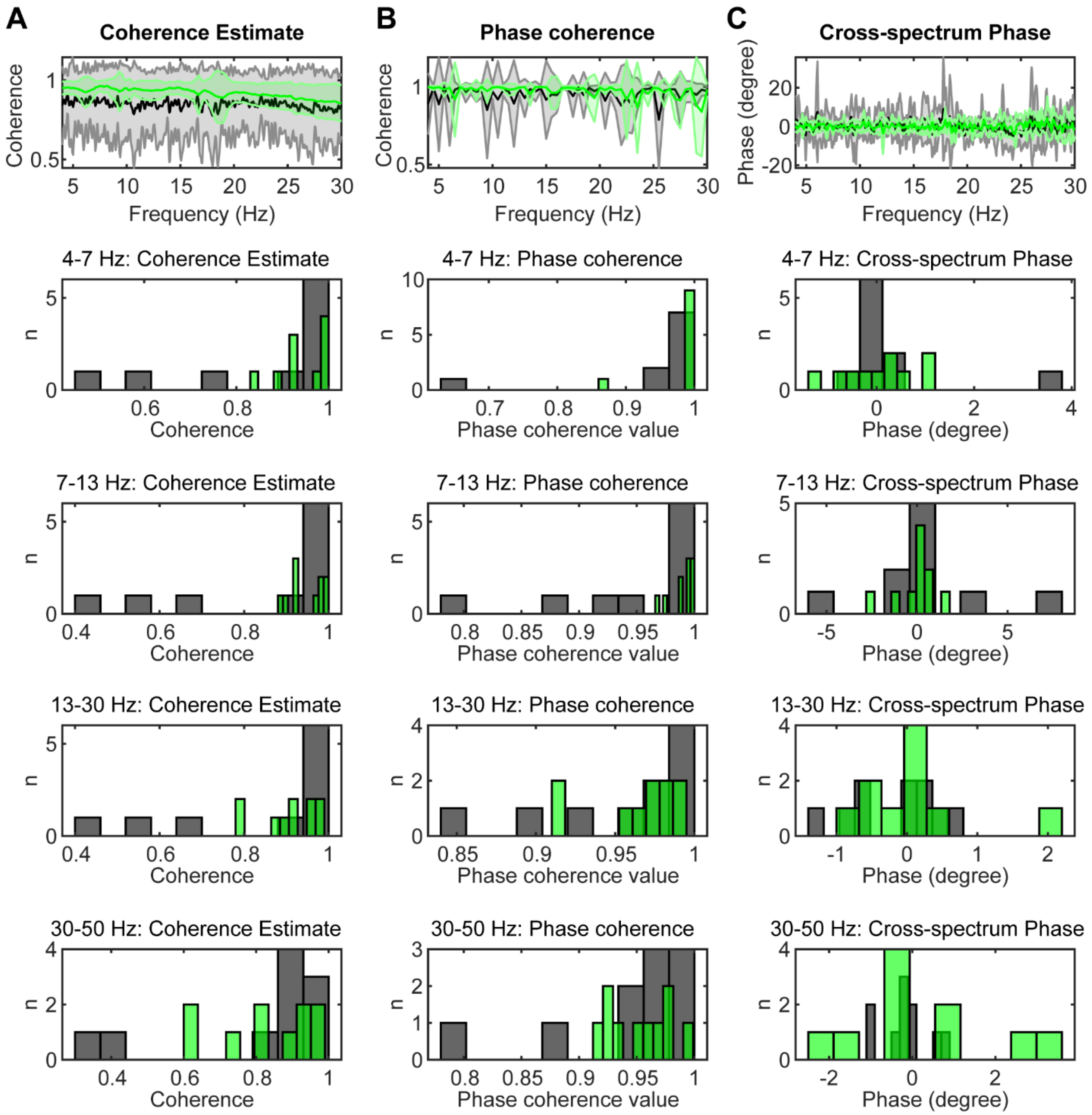

Fig. S4. Similarity analyses between mid-cerebellar 'Cbz' and mid-occipital 'Oz' signals. Coherence estimates (A), phase coherence (B), and cross-spectrum phase (C) were compared across frequency bands including delta, theta, alpha, beta, and gamma frequency bands.
